# Relationship between Interlimb Asymmetries and Performance Variables in Adolescent Tennis Players

**DOI:** 10.3390/life14080959

**Published:** 2024-07-30

**Authors:** Oscar Villanueva-Guerrero, Héctor Gadea-Uribarri, Víctor Emilio Villavicencio Álvarez, Santiago Calero-Morales, Elena Mainer-Pardos

**Affiliations:** 1 Health Sciences Faculty, San Jorge University, Autov. A23 Km 299, Villanueva de Gállego, 50830 Zaragoza, Spain; ovillanueva@usj.es (O.V.-G.); epardos@usj.es (E.M.-P.); 2 Faculty of Education, Pontifical University of Salamanca, 37002 Salamanca, Spain; hgadeauribarri@hotmail.com; 3 Department of Human and Social Sciences, Armed Forces University-ESPE, Quito 171103, Ecuador; victoremiliovillavicencio@gmail.com

**Keywords:** adolescent, tennis, multidirectional speed, performance, asymmetry

## Abstract

Tennis is an individual sport characterised by high-intensity action, including sprints and changes of direction (COD). However, there is a gap in the knowledge of limb asymmetries in adolescent tennis players and their association with performance. The aim of this study was to investigate the relationship between asymmetry in vertical and horizontal jump tests (CMJ and HJ) and COD with lower limb performance variables in adolescent tennis players. Forty-five adolescent tennis players (age: 13.81 ± 1.08 years; height: 167.64 ± 9.9 cm; body mass: 57.48 ± 10.94 kg; body mass index: 20.27 ± 2.18 kg/m^2^) performed the CMJ test (unilateral and bilateral), horizontal jumps (unilateral and bilateral), 25 m sprint, and 180° COD test. The single-leg countermovement jump showed the greatest asymmetries among the different tests (6.62 ± 9.35%). Notable negative relationships were found between CMJ asymmetry and COD asymmetry with unilateral horizontal jump variables (*r* = −0.30 to −0.53). In addition, CMJ asymmetry showed notable relationships with CMJR (*r* = 0.49) and COD180R (*r* = 0.29), whereas COD asymmetry showed a notable relationship with COD180L (*r* = 0.40). On the other hand, HJ asymmetry showed no notable relationships with any variable. The main findings of this study indicate that greater imbalances in the CMJ and COD tests are associated with a reduced capacity to perform unilateral horizontal jumps. These results suggest the incorporation of training protocols to reduce interlimb asymmetries in growing athletes to improve their performance. This practical application is important for coaches and physical trainers who aim to improve the physical performance of adolescent tennis players. Greater asymmetry results in a reduced ability to produce unilateral horizontal force.

## 1. Introduction

Tennis is a sport in which individuals or pairs compete to hit the ball over the net so that the opponent cannot return the shot [1]. The court measures 11.90 × 10.97 m [2], and points can last from 4 to 10 s, with breaks of 10 to 20 s between points [1]. This sport involves high-intensity actions such as sprints (between 8 m and 15 m), accelerations, decelerations, changes of direction (3–4 on average), and jumps [1,2,3,4,5]. It is very unlikely that these actions will occur the same number of times on each limb, making it highly likely that neuromuscular asymmetries will develop over the course of the season (5).

Limb asymmetry has been a common line of research in recent years [3,6]. The first study to analyse the impact of asymmetries on performance was in 2018 by Coratella et al. [7]. This term refers to the differences between one limb and the other [8,9]. These asymmetries indicate strength deficits between the two limbs [3]. For example, the ratio of hamstrings to quadriceps (H:Q) is between 50% and 80%. In addition, asymmetry greater than 10–15% is associated with a higher risk of injury [3,10,11]. In tennis, there is a paucity of literature on asymmetry [2,12]. Therefore, it is important for sports coaches to understand the importance of limb asymmetry to improve the performance of their athletes [11].

Limb asymmetries and their association with injury risk have been the subject of increasing research in recent years [10,13,14]. The lower limbs are most commonly injured (39–59%), followed by the upper limbs (20–40%) and the trunk (11–30%) [15,16]. Asymmetries between 10% and 15% are often associated with a higher risk of injury and reduced performance [6,17]. If these tests reveal an asymmetry of >10%, this indicates a fourfold increase in the risk of lower limb injury. Similarly, an asymmetry of more than 4 cm on the Y-balance test (YBT) would indicate the same increased risk of injury [18]. Therefore, strength training programmes should include both bilateral and unilateral exercises to reduce these asymmetries [8]. Gonzalo-Skok et al. suggest training the weaker leg first, with the same or even double the volume of the weaker leg compared to the stronger leg to improve performance and reduce asymmetry [19].

Finally, various tests are used to detect asymmetries in athletes using jump tests. The single-leg vertical and horizontal jump are reliable and efficient methods [6,14]. Other tests used include measuring the isokinetic peak for knee flexion and extension between the dominant and non-dominant limbs [10]. Studies have shown that once the differences between the limbs have been identified, the implementation of a neuromuscular training programme can reduce these asymmetries [2].

Furthermore, the relationship between asymmetries and performance has increased the number of publications on this topic recently. In general, strength asymmetry is negatively correlated with jump performance, speed, and change of direction speed [6]. Other studies have shown that athletes with better symmetry data are faster than asymmetric athletes in team sports [8]. Another study suggests that greater lower limb asymmetry in jumping tests is associated with a reduction in 5, 10, and 20 m sprints in youth soccer players [5]. A final study suggests that asymmetries in the one-legged vertical jump test are associated with slower 5 m sprint speeds in youth soccer players [20]. Performance and asymmetries have been analysed in many team sports. However, they have not been analysed in individual sports, so more research is needed before conclusions can be drawn.

Despite the growing research in the field of asymmetries and their relationship with physical performance, there is a notable gap in knowledge regarding how these asymmetries affect specific performance variables. This is particularly true for 180° changes of direction (COD), especially in adolescent populations who are still growing up. This study aims to fill this gap by investigating the relationship between limb asymmetries and physical performance variables in adolescent tennis players. This will provide important information for optimising training programmes and reducing the risk of injury in this population. Therefore, the main objective of this study was to examine the relationship between asymmetry in vertical and horizontal jump tests (CMJ and HJ) and COD with lower limb performance variables in adolescent tennis players. This study hypothesises that interlimb asymmetries in CMJ, HJ and COD are negatively correlated with lower limb performance variables in adolescent tennis players.

## 2. Materials and Methods

### 2.1. Participants

Forty-five trained [21] adolescent male tennis players (age: 13.81 ± 1.08 years; height: 167.64 ± 9.9 cm; body mass: 57.48 ± 10.94 kg; body mass index: 20.27 ± 2.18 kg/m^2^) consented to participate in the study. An initial power analysis was conducted to determine the necessary number of participants using the G*Power software (version 3.1.9.3, based in Düsseldorf, Germany). Considering the study design, which investigates variances within a single group and accounts for an effect size of 0.5, a notable level (alpha) of 0.05, and a desired power of 95%, it was established that 38 subjects would be required. Nonetheless, the study included 45 participants, giving a statistical power of 97%.

All participants were competitive tennis players who regularly participated in regional and national tournaments. Their competitive level ranged from intermediate to advanced, and some players were in the top 100 of their respective age categories in the national rankings.

All tennis players belonged to two different tennis academies and followed a systematically structured training programme, consistent in both volume and methodology. This training programme included four 90 min technical-tactical training sessions per week. These sessions focused on the development of specific tennis skills such as serving, volleying, baseline play, doubles strategies, and so on. Two 60 min physical training sessions per week. Physical training included strength and conditioning exercises designed to improve muscular strength, endurance, balance, COD, and/or flexibility. Exercises included bilateral and unilateral movements, plyometric exercises, sprint intervals, and sport-specific conditioning. The intensity and volume of training sessions vary according to the proximity of the players to the competition and are adjusted to optimise performance and recovery. When players are far from competition, training phases involve a higher load and intensity. As the competition approaches, the intensity and volume of training sessions are adjusted to ensure the transfer of these adaptations to tennis performance. The training programme also included specific drills to improve singles and doubles performance, emphasising coordination, communication, and strategy. Skill development sessions focused on improving technique, stroke accuracy, and consistency. Tactical training that incorporated match simulations, strategic play analysis, and mental conditioning exercises to improve concentration and decision-making under pressure.

The players had an average of at least 5 years of experience playing tennis, having started training at an early age. They had been competing at the regional and national levels for an average of at least 3 years.

To minimise selection bias, participants were drawn from two different tennis academies with similar training volumes and methodologies. This ensured a diverse representation of competitive adolescent tennis players.

In addition, the players were injury-free during the data collection period. Written informed consent was obtained from the parents or guardians of all participants. The study was conducted in accordance with the ethical principles outlined in the Declaration of Helsinki (2013) and received approval from the University Ethics Committee (approval no. 46/2/22–23).

### 2.2. Procedures

Physical performance assessments were conducted on the same day at the players’ usual training times (18:00) in favourable weather conditions. Players were instructed to avoid strenuous physical activity for 48 h before the assessment tests. All players were familiar with the tests to be conducted, as these are tests that are included in the Academy’s programme throughout the season. Players tested on a hard tennis surface using specific tennis shoes. A Rise, Activate, Mobilise, and Potentiate (RAMP) system warm-up protocol was performed before testing [22]. After the warm-up, three practice runs were completed for each test. A 3 min rest period was allowed between the last practice run and the start of the first test. The order in which the tests were performed was as follows: unilateral vertical jumps, bilateral vertical jumps, unilateral horizontal jumps, bilateral horizontal jumps, 180° COD, and 25 m sprint. Unilateral tests were always started with the right leg and finished with the left leg. There was a 3 min break between races to allow players to hydrate.

#### 2.2.1. Unilateral and Bilateral Countermovement Jump Test

Lower body performance in the vertical plane was measured using the countermovement jump test. Optogait systems (Witty, Microgate, Bolzano, Italy) were used as an evaluation instrument to measure performance (jump height), using the protocol described by Pardos-Mainer et al. [23]: Players then completed three practice jumps to familiarise themselves with the procedure. Players were instructed to keep their hands on their hips during the entire jump to prevent the use of arm swings. Without bending their legs during the flight phase, players performed a maximal vertical jump.

The evaluation protocol for each test (bilateral, right, and left) was performed twice, with 45 s of recovery between each attempt. The best score in each test was recorded for subsequent analysis using the following variables: countermovement jump right (CMJR), countermovement jump left (CMJL), and bilateral countermovement jump (CMJ). The reliability of these measurements was confirmed by an Intraclass Correlation Coefficient (ICC) of 0.82 to 0.86.

#### 2.2.2. The Unilateral and Bilateral Horizontal Jump Test

Lower body performance in the horizontal plane was measured using the horizontal jump test of tennis players. Standard 30 m tape measure 30 m (M13; Stanley, New Britain, EEUU) was used as an evaluation instrument to measure performance (distance), using the protocol described by Pardos-Mainer et al. [23]: Players then completed three practice jumps to familiarise themselves with the procedure. Each tennis player stood behind a starting line marked with tape, hands relaxed at their sides. When ready, each player performed a jump as far as possible, landing while maintaining balance for 2–3 s.

The evaluation protocol for each test (bilateral, right, and left) was performed twice, with 45 s of recovery between repetitions. The longest jump distance from each test was recorded for subsequent analysis using the following variables: horizontal jump right (HJR), horizontal jump left (HJL), and bilateral horizontal jump (HJ). The intraclass correlation coefficients (ICC) achieved ranged from 0.87 to 0.90 for these tests.

#### 2.2.3. The 25 m Sprint Test

The 25 m sprint tests were conducted to assess the top speed of the tennis players. The timing was performed using double-beam photoelectric cells (Witty, Microgate, Bolzano, Italy). The timing gates were placed 1.5 m apart and at a height of 0.75 m. Each tennis player positioned himself behind the starting line, which was 0.5 m before the first marker. The players then completed two practice sprints to familiarise themselves with the procedure. Players started the sprint at their own discretion without any external signals. The total sprint distance was 25 m. Two repetitions of this test were performed. There was a recovery period of 2 min between each attempt to prevent fatigue. The fastest time of the two sprints was selected for subsequent statistical analysis. The reliability of this measurement was confirmed by an ICC of 0.94.

#### 2.2.4. The 180° COD Test

The change of direction performance was performed using a 10 m sprint with a COD of 180°. Photoelectric cells (Witty, Microgate, Bolzano, Italy) were used to measure the performance (i.e., time) using the protocol described by Pardos-Mainer et al. [23]: Photoelectric cells were positioned to measure the start and finish times. Each tennis player sprinted from the start line, crossed the 5 m line completely with either the right or left foot, and then turned 180° to sprint back to the finish line. Four COD tests were performed by each player: two using the right leg and two using the left leg. There was a recovery period of 2 min between repetitions to prevent fatigue.

The shortest time with each leg was recorded for statistical analysis with the following variables: 10 m sprint with one change of direction to the right (COD180R) and 10 m sprint with one change of direction to the left (COD180L). The reliability of this test was confirmed by an ICC of 0.95.

### 2.3. Statistical Analyses

SPSS statistical software (Version 28.0; SPSS Inc., Chicago, IL, USA) was used for statistical analysis. The Shapiro–Wilk test was performed to assess normality for all variables.

First, a one-way repeated measures ANOVA was performed to detect any systematic bias between the means of asymmetry in CMJ, HJ, and COD performance variables. Asymmetries between the lower limbs were expressed as percentages (%) using the following equation [24]:

100/Max value (right and left) ∗ Min value (right and left) ∗ −1 + 100

Pearson’s correlation test was used to determine correlations between limb asymmetries (%) and physical performance. Based on Hopkins et al., the scale of correlation coefficients was classified as follows: trivial for *r* less than 0.01, small for *r* from 0.1 to less than 0.3, moderate for *r* from 0.3 to less than 0.5, large for *r* from 0.5 to less than 0.7, numerous for *r* from 0.7 to less than 0.9, nearly perfect for *r* from 0.9 to less than 1, and perfect when *r* equalled 1 [25]. A value of 0.05 was considered statistically notable. In addition, the kappa coefficient was used to measure the consistency of the direction of asymmetry between tests, interpreted as follows: poor (≤0), slight (0.01–0.20), fair (0.21–0.40), moderate (0.41–0.60), substantial (0.61–0.80), almost perfect (0.81–0.99), and perfect [26].

## 3. Results

Descriptive data on the physical performance and asymmetries of tennis players are presented in Table 1. After comparing the mean asymmetry of the groups, the ANOVA test showed no statistically significant results.

Table 2 presents Pearson’s correlations between interlimb asymmetry scores and performance tests. Notable correlations were observed between CMJ Asymmetry and the variables CMJR (*p* = 0.01; *r* = −0.49; ES = 0.24), HJR (*p* = 0.01; *r* = −0.53; ES = 0.28), HJL (*p* = 0.01; *r* = −0.52; ES = 0.27) and COD180R (*p* = 0.05; *r* = 0.29; ES = 0.08). The Hopkins scale classified the correlations of CMJ Asymmetry with unilateral horizontal jumps as numerous, with CMJR as large and with CODR as moderate. Moreover, notable correlations were identified between COD Asymmetry and the variables HJR (*p* = 0.04; *r* = −0.30 ES = 0.09), HJL (*p* = 0.01; *r* = −0.38; ES = 0.14), and COD180L (*p* = 0.01; *r* = 0.40 ES = 0.16). The Hopkins scale classified the correlations of COD Asymmetry with unilateral horizontal jumps and with CODL as large. Conversely, no notable relationships were found between HJ Asymmetry and the variables associated with jumping, speed, and COD.

**Figure 1:**
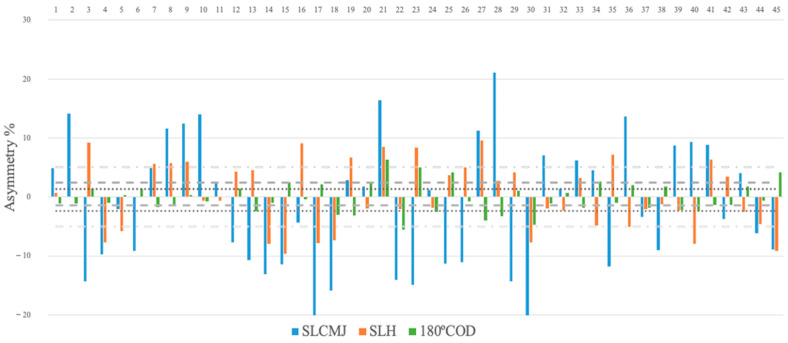
Individual asymmetry data for countermovement jump (CMJ), horizontal jump (HJ) and 180° change of direction (180° COD). Note: Values above 0 indicate right leg dominance and values below 0 indicate left leg dominance.

Table 3 shows the levels of agreement of the asymmetry scores as measured by the Kappa coefficient. The results indicate fair agreement between the CMJ test and the 180° COD test (−0.15) and slight agreement between the CMJ and HJ tests (0.01) and between the HJ and COD180 tests (−0.06). Figure 1 illustrates the discrepancies between the limbs for CMJ, HJ, and COD, highlighting the variability in both the magnitude and direction of the asymmetry.

## 4. Discussion

The main findings of this study indicate notable correlations between the asymmetries observed in vertical jump and COD with single-leg horizontal jump (SLHJ) performance in highly trained adolescent tennis players. These correlations suggest that greater imbalances in the CMJ and COD tests are associated with a reduced ability to perform unilateral horizontal jumps, both with the right leg (HJR) and the left leg (HJL). In addition, asymmetries between jump and COD tests rarely favoured the same side, indicating the task-specific nature of the asymmetry. Previous studies have used different metrics to assess concordance between performance tests [2], which could provide a more direct comparison of our findings.

The single-leg countermovement jump (SLCMJ) showed the greatest asymmetries among the different tests (6.62 ± 9.35%), as shown in previous studies with tennis players (15.03 ± 6.91%) [2]. CMJ asymmetry has a notable correlation with the right leg vertical jump (*r* = −0.49), according to the study by Bishop et al., who found the same notable correlation for the right leg (*r* = −0.47) and the left leg (*r* = −0.53) [27]. In addition, there was a notable correlation with unilateral horizontal jumps in both the right and left legs. Regarding 180° COD, there is a notable correlation with the one performed using the right leg, which differs from the literature [6]. These results contradict another study of tennis players, which found a relationship between greater asymmetry and lower performance in COD [2]. However, in a handball article by Madruga et al. [28], there is a notable correlation with the 8 × 10 m repeated sprint test. Coratella et al. [7] correlated 10 m and 30 m sprint times moderately and positively with high-speed concentric peak torque asymmetry between the hamstrings. These differences may be due to the specific demands and movement patterns of tennis. Asymmetries can result from unilateral training and the predominant use of one limb. For strength and conditioning coaches, these differences have important practical applications. They need to develop training programmes that minimise asymmetries and enhance the strength and agility of both legs. This approach improves performance and reduces the risk of injury [19].

The asymmetries in the single-leg horizontal jump (3.97 ± 4.18%) are similar to previous studies in tennis players (4.14 ± 3.72%) [2] and team sport athletes (3.3 ± 3.0%) [29]. In HJ asymmetry, there is no notable correlation with any variable, which is consistent with studies such as Bishop et al. [30]. However, Roso-Moliner et al. [31] found a notable correlation with CMJ asymmetry, which is greater in the left leg than in the right leg, as well as with HJ in the right leg of female football players. This may be because there are more vertical jumps in soccer than in tennis. This highlights the importance of future studies comparing different sports that work at different levels. In the present study, no significant relationships were found between HJ asymmetry and performance variables. This could indicate that, for adolescent tennis players, HJ asymmetries do not have a considerable impact on performance. Alternatively, it might suggest that other factors, not measured in this study, could be influencing performance. This could be due to the specific nature of the movement in tennis, where unilateral actions predominate. It is possible that the HJ tests do not fully capture the specific demands of tennis, suggesting the need to develop more sport-specific tests. On the other hand, strength and conditioning coaches should programme training in different planes and include both bilateral and unilateral training [32].

The asymmetries in COD (1.47 ± 2.07%) were smaller than the asymmetries of both jumps (6.62 ± 9.35%) and (3.97 ± 4.18%), showing similarity with the existing literature (1.83 ± 1.43%) [2] and (2.39 ± 1.64%) [6]. COD asymmetry in relation to HJ showed a notable correlation for both the right and left legs, with the left leg showing a stronger correlation. For 180° COD, the notable correlation with the left leg was positive, suggesting that players with greater asymmetry are more efficient at changing direction with this leg. However, in Madruga et al. [2] on tennis players, a notable correlation with the SLCMJ was observed. These observations suggest that asymmetries may differentially affect performance in different tests and skills. It is interesting to perform different tests based on specific actions of each sport analysed to understand the asymmetries of athletes [18,32].

Regarding the analysis of the Kappa results, it should be noted that in the present article, in the SLCMJ, there are 20 players with above 10% asymmetry. In both SLH and 180° COD, all athletes had below 10% asymmetry. These data are very similar to the handball article by Madruga et al. [28], where 21 players have above 10% asymmetry in the SLCMJ. In another article on women’s soccer by Roso-Moliner et al., only six players have >10% asymmetry in the SLCMJ and none in the SLHJ [31]. This means that the kappa can underestimate or overestimate the agreement if the magnitude of the differences is relevant to the performance. In all cases, the strength-and-conditioning coach should implement a training protocol to correct these asymmetries. This will increase athlete performance and reduce the risk of injury by keeping asymmetries below 10% [28,33].

While these findings are valuable, the study has some limitations. First, only males were included in this study, which may limit the generalisability of the results to female tennis players. However, this methodology was used due to the availability and access to a homogenous group of adolescent male tennis players, which allows for a more direct and controlled comparison of asymmetries and physical performance. Second, only non-professional athletes, not professional athletes, were included. This choice was based on the intention to study players at important stages of their physical performance development, where asymmetries may have a greater impact on performance and injury risk. Third, this study was conducted with 14-year-old athletes, and it would be interesting to conduct a study with players at different stages of maturation. This specific age was chosen because it is an important stage in adolescent development, as well as providing novel information at a stage of growth prior to the study by Madruga et al. [2]. Finally, the current study has some limitations due to unmeasured confounding variables. Factors such as fatigue, the type of playing surface, and differences in training techniques could have influenced the results.

Differences in asymmetries and their relationship to physical performance have important practical applications for coaches and trainers. It is essential to develop training programmes that minimise asymmetries and improve the strength and COD of tennis players. Programmes should include both bilateral and unilateral exercises, depending on the asymmetries of each player. They should be tailored to the individual needs of each tennis player to maximise performance and reduce the risk of injury.

For future research, a systematic review of asymmetries in different sports and levels of competition is recommended to better understand how they affect performance and injury risk. In addition, it would be valuable to investigate differences between male and female players, as well as between athletes of different ages and stages of maturation.

## 5. Conclusions

This study analysed lower limb asymmetries in relation to performance variables in adolescent tennis players. In summary, asymmetries found in the CMJ and 180° COD were related to lower performance in unilateral horizontal jump tests with both the right and left legs. However, HJ asymmetry did not show a notable relationship with any performance variable. Due to the association of asymmetries with reduced physical performance, it is necessary to adapt training programmes to reduce asymmetries between limbs. The direction of asymmetries varies greatly in tennis players; therefore, it is necessary to individualise training for each subject to reduce asymmetries and improve their performance, such as following the study by Gonzalo-Skok et al., starting the session with the weaker leg and doing twice the sets compared to the stronger leg [19].

## Figures and Tables

**Table 1:** Descriptive data for all physical performance tests.

Variable	Mean ± SD	*p* (ES)	Asymmetry (%)
CMJR (cm)	14.46 ± 3.31	0.17 (0.04)	6.62 ± 9.35
CMJL (cm)	14.82 ± 3.70		
HJR (cm)	153.42 ± 21.90	0.93 (0.01)	3.97 ± 4.18
HJL (cm)	153.53 ± 24.24		
COD180R (s)	2.85 ± 0.12	0.58 (0.01)	1.47 ± 2.07
COD180L (s)	2.86 ± 0.12		
CMJ (cm)	29.11 ± 5.05		
HJ (cm)	190.38 ± 24.44		
20 m (s)	3.41 ± 0.17		

CMJR and CMJL: unilateral countermovement jump with right and left legs; HJR and HJL: unilateral horizontal jump with right and left legs; COD180R and COD180L: 10 m shuttle sprint with one change of direction to right or left; CMJ: bilateral countermovement jump; HJ: bilateral horizontal; jump20 m: linear sprint of 20 m; ES: Effect Size.

**Table 2:** Correlations between jump and change of direction tests and asymmetry percentages.

Test	CMJ Asymmetry	*p*	ES (*r*^2^)	HJ Asymmetry	*p*	ES (*r*^2^)	COD Asymmetry	*p*	ES (*r*^2^)
CMJR (cm)	−0.49 **	0.01	0.24	−0.08	0.58	0.01	−0.15	0.31	0.02
CMJL (cm)	−0.17	0.24	0.03	0.03	0.81	0.01	−0.05	0.70	0.01
HJR (cm)	−0.53 **	0.01	0.28	−0.28	0.06	0.07	−0.30 *	0.04	0.09
HJL (cm)	−0.52 **	0.01	0.27	−0.17	0.26	0.02	−0.38 **	0.01	0.14
COD180R (s)	0.29 *	0.05	0.08	0.24	0.10	0.05	0.25	0.08	0.06
COD180L (s)	0.27	0.12	0.07	0.12	0.40	0.01	0.40 **	0.01	0.16
CMJ (cm)	−0.28	0.06	0.08	−0.06	0.66	0.01	−0.06	0.69	0.01
HJ (cm)	−0.22	0.14	0.05	0.07	0.61	0.01	0.00	0.99	0
20 m (s)	−0.25	0.9	0.06	−0.28	0.06	0.07	−0.16	0.27	0.02

CMJR and CMJL: unilateral countermovement jump with right and left legs; HJR and HJL: unilateral horizontal jump with right and left legs; COD180R and COD180L: 10 m shuttle sprint with one change of direction to right or left; CMJ: bilateral countermovement jump; HJ: bilateral horizontal; jump20 m: linear sprint of 20 m. * Correlation is notable at the 0.05 level (bilateral). ** Correlation is notable at the 0.01 level (bilateral). ES: Effect Size.

**Table 3:** Descriptive levels of agreement and Kappa coefficients for asymmetries between jumping, speed, and COD tests.

Test	Kappa Coefficient	Descriptor
CMJ-HJ	0.02	Slight
CMJ-COD180	−0.15	Fair
HJ-COD180	−0.06	Slight

CMJR and CMJL: unilateral countermovement jump with right and left legs; HJR and HJL: unilateral horizontal jump with right and left legs; COD180R and COD180L: 10 m shuttle sprint with one change of direction to right or left; CMJ: bilateral countermovement jump; HJ: bilateral horizontal; jump20 m: linear sprint of 20 m.

## Data Availability

The data from this research can be made available by the corresponding author following a justified request. Due to privacy concerns, the data are not accessible to the public.
